# Epidemiological and Etiological Characteristics of Hand, Foot, and Mouth Disease in Henan, China, 2008–2013

**DOI:** 10.1038/srep08904

**Published:** 2015-03-10

**Authors:** Xueyong Huang, Haiyan Wei, Shuyu Wu, Yanhua Du, Licheng Liu, Jia Su, Yuling Xu, Haifeng Wang, Xingle Li, Yanxia Wang, Guohua Liu, Weijun Chen, John David Klena, Bianli Xu

**Affiliations:** 1 Henan Center for Disease Control and Prevention, Zhengzhou, People's Republic of China; 2 Henan Key Laboratory of pathogenic microorganisms, Zhengzhou, People's Republic of China; 3 International Emerging Infections Program, US Centers for Disease Control and Prevention, Beijing, People's Republic of China; 4 Global Disease Detection Branch, Division of Global Health Protection, Center for Global Health, Centers for Disease Control and Prevention, Atlanta, USA; 5 State Key Laboratory of Pathogens and Biosecurity, Institute of Microbiology and Epidemiology, Academy of Military Medical Sciences, Beijing, People's Republic of China; 6 Key Laboratory of Genome Sciences and Information, Beijing Institute of Genomics, Chinese Academy of Sciences, Beijing, People's Republic of China

## Abstract

Hand, foot, and mouth disease (HFMD) is a common childhood illness caused by enteroviruses. HFMD outbreaks and reported cases have sharply increased in China since 2008. Epidemiological and clinical data of HFMD cases reported in Henan Province were collected from 2008 to 2013. Clinical specimens were obtained from a subset of these cases. Descriptive epidemiological methods were used to analyze the time, region and population distribution. The VP1 gene from EV71 and CA16 isolates was amplified, and the sequences were analyzed. 400,264 cases of HFMD were reported in this study, including 22,309 severe and 141 fatal cases. Incidence peaked between April and May. Laboratory confirmation was obtained for 27,692 (6.9%) cases; EV71, CA16, and other enteroviruses accounted for 59.5%, 14.1%, 26.4%, respectively. Phylogenetic analysis revealed that EV71 belonged to the C4a evolution branch of C4 sub-genotype and CA16 belonged to subtype B1a or B1b. The occurrence of HFMD in Henan was closely related to season, age and region distribution. Children under five were the most affected population. The major pathogens causing HFMD and their genotypes have not notably changed in Henan. The data strongly support the importance of EV71 vaccination in a high population density area such as Henan, China.

Hand, foot, and mouth disease (HFMD) is a common infectious disease of global concern mainly occurring in children aged < 5 years. Since the 1980s, multiple severe HFMD outbreaks have been documented and HFMD remains a significant public health challenge, especially in the Asia-Pacific region^1^,^2^,^3^,^4^,^5^,^6^. In China, incidence of reported cases of HFMD has sharply increased and more than 7 million cases have been reported, including approximately 2500 fatal cases, since the initiation of national surveillance for HFMD in 2008^7^,^8^,^9^,^10^. HFMD is caused by a spectrum of pathogens in the enterovirus (EV) family, but human enterovirus 71 (EV71) and coxsackievirus A16 (CA16) are common etiological agents; most patients with fatal complications are infected with EV71^11^,^12^. Transmission of HFMD occurs from person-to-person via direct contact with nasal discharge, saliva, vesicular fluid from an infectious person, or faecal-oral route facilitating rapid spread of the disease in a community^13^,^14^. No effective chemoprophylaxis or vaccination is available for controlling HFMD. The epidemic of HFMD is considered a serious public health and social problem in China^9^,^15^,^16^.

Henan Province is located in eastern central China and divided into 18 cities/counties. The province consists of central and eastern plains; a southwestern basin; northern, western and southern mountain ranges; and a territory lying tilted from west to east. Henan is one of the provinces most affected by HFMD in China. Increases in reported cases in all cities/counties of Henan Province began in 2008 and in 2009, a total of 101,480 cases were reported with an incidence rate of 107.63 per 100,000 population, 3^rd^ among 31 provinces in China^17^. The proportion of severe cases (defined as having infection accompanied by neurological impairment, respiratory disorder or circulatory disturbance) in Henan Province was markedly higher than the national average, and the number of severe cases was highest among China's 31 provinces from 2009 to 2013.

Using surveillance data of HFMD collected from the national reportable disease surveillance system in China^18^, the epidemiological and etiological characteristics of HFMD in Henan Province from January 2008 to December 2013 were explored. The goal of this study was to provide information to help guide the development of effective prevention and control measures in Henan province.

## Results

### Epidemiology features

#### Demographic characteristics

From 1 January 2008 to 31 December 2013, a total of 400,264 HFMD cases were reported in Henan Province, including 22,309 severe and 141 fatal cases. The annual average incidence rate of HFMD was 70.87 per 100,000 (range: 1.63 to 107.63 per 100,000). Among these cases, 256,230 were males and 144,034 were females, with an average male-to-female sex ratio 1.78 (range: 1.62 to 1.99). The annual average incidence in males (88.25 per 100,000) was higher than in females (52.49 per 100,000) and this difference was statistically significant (χ^2^ = 196.19, p < 0.0001). The majority (82%) of cases occurred in scattered children^19^. The percentage of cases who attended a kindergarten or other school was 16% and 2%, respectively (Table 1).

The median age of the 400,264 HFMD cases was 2 years (range, 1 month to 70 years). There was a statistically significant difference with respect to HFMD cases among the five age groups (χ^2^ = 2874.53, p < 0.0001); Most [65.5% (254,270/400,264)] cases were in the 1- < 3 year age group; 99.3% of cases were aged < 10 years. The majority of cases detected had mild to moderate symptoms (94.4%, 377,814/400,264); the remaining cases were classed as severe with 0.63% of cases resulting in death. The highest percentage of severe cases (6.6%) was observed in the 1- < 3 year group, while children aged < 1 year were associated with the greatest percentage of case fatalities (0.06%, 20/33,855) (Table 1).

#### Seasonality

**Figure 1:**
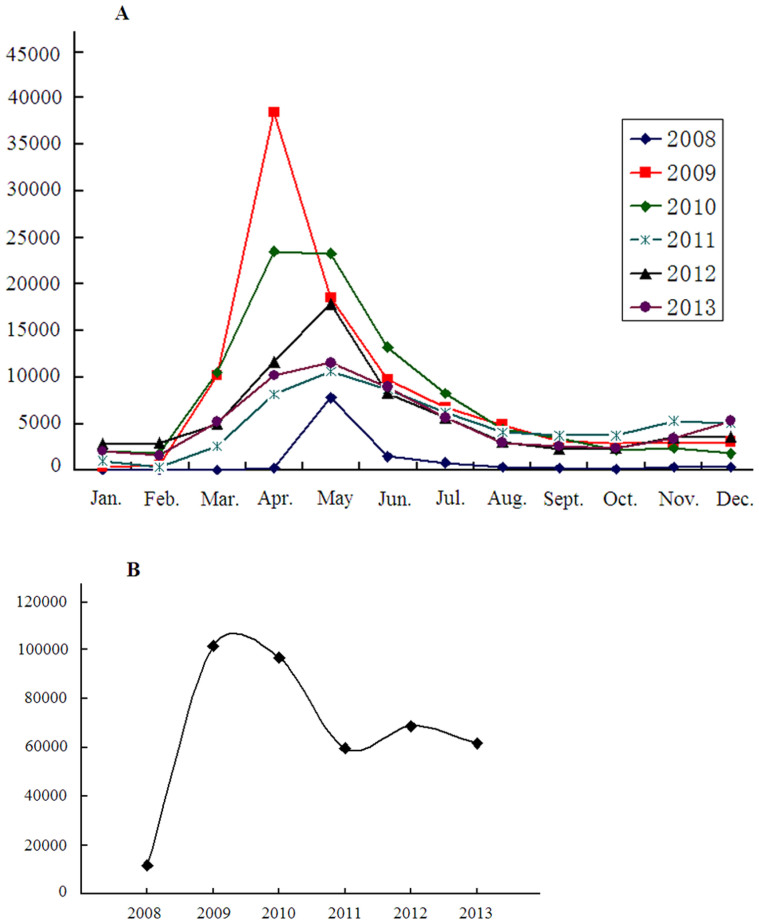
Monthly (A) and Yearly (B) distribution of HFMD cases, Henan Province, 2008 to 2013.

Although HFMD cases occurred throughout the year, an increase in case reporting over background was evident from February to June; peaks were seen in April (2009 and 2010) and May (all other years). The annual number of HFMD cases was lowest (11,826) in 2008 and greatest (101,480) in 2009; from 2011 to 2013 cases were remained elevated from 2008 but stable (Figure 1).

#### HFMD case clusters

There were 4,575 reports of HFMD case clusters through cluster reporting system in Henan from 2008 to 2013, involving a total of 17,474 cases. The majority of HFMD case clusters occurred in villages and Kindergartens, accounting for 70.1% (3,233/4,575) of all reports (Table 1). HFMD case clusters occurred mainly between April and June; this was consistent with the time distribution of sporadic cases.

#### Location distribution

**Figure 2:**
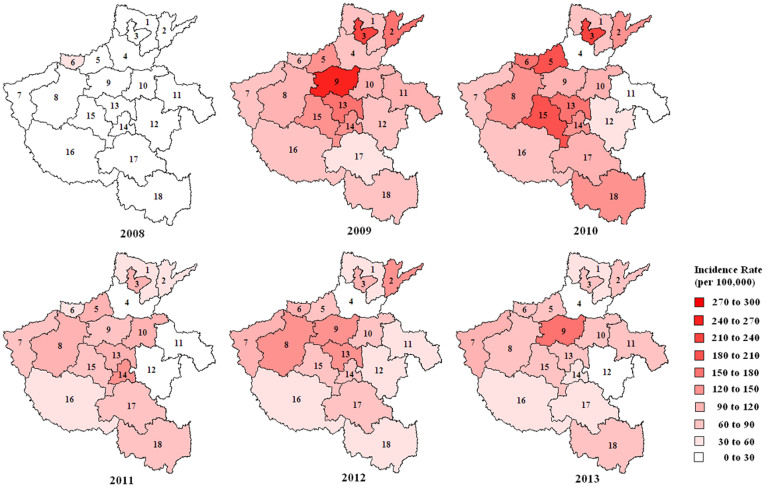
Annual HFMD incidence rates at the provincial municipality level, Henan Province, 2008 to 2013. Numbers indicate the following municipalities: 1. Anyang, 2. Puyang, 3. Hebi, 4. Xinxiang, 5. Jiaozuo, 6. Jiyuan, 7. Sanmenxia, 8. Luoyang, 9. Zhengzhou, 10. Kaifeng, 11. Shangqiu, 12. Zhoukou, 13. Xuchang, 14. Luohe, 15. Pingdingshan, 16. Nanyang, 17. Zhumadian, 18. Xinyang. Figure was made using the PHGIS software version 1.02.

Clear differences in the annual incidence of HFMD among provincial cities were observed. In 2008, the incidence of HFMD was less than 30 per 100,000 population in Henan Province except for Jiyuan municipality (53.83 per 100,000). In 2009–2010, high incidence rates were mostly found in the center of the study area, however, the epidemic quickly spread, particularly into the north and south, and HFMD cases were reported in all provincial municipalities of Henan. From 2011 to 2013, incidence was relatively low, especially in Xinxiang, Shangqiu and Zhoukou cities (Figure 2).

#### Space-time clusters

**Figure 3:**
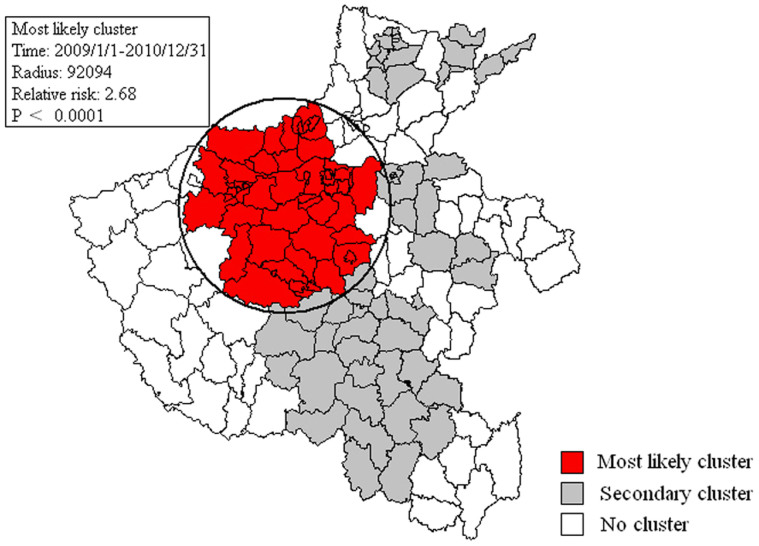
Detected spatial-temporal clusters of HFMD, Henan Province, 2008 to 2013. Figure was made using the PHGIS software version 1.02.

Using space-time scan statistics based on the Space-Time discrete Poisson model, statistically significant spatial-temporal clusters for a high occurrence of HFMD during 2008 to 2013 were detected. Five spatial-temporal clusters were detected from 2008 to 2013, including one most likely cluster and four secondary clusters, meaning less likely to be a real cluster (Supplementary Table 2 and Figure 3). The most likely cluster consisted of 78,431 cases and was located in northern central Henan Province from January 2009 to December 2010; 54 county townships were affected. Secondary clusters were scattered in the northern, eastern and southern districts. The largest secondary cluster was located in southern Henan from January 2010 to December 2010; 26 county townships were affected.

### Laboratory detection

Of the 400,264 reported HFMD cases, 27,692 (6.9%) were laboratory confirmed, with 16,484 (59.5%), 3,914 (14.1%), and 7,294 (26.4%) associated with EV71, CA16, and other enteroviruses, respectively. There were statistical differences in the rate of EV71, CA16 and other enteroviruses detected during different study years (χ^2^ = 1331.17, *P* < 0.0001). Samples were positive for EV71 more often than CA16 or other enteroviruses (χ^2^ = 966.56 or 513.68, *P* < 0.0001, respectively), and the positive of EV71 more than CA16 or other enteroviruses in mild cases (χ^2^ = 340.54 or 264.99, *P* < 0.0001, respectively) and severe cases (χ^2^ = 127.25 or 89.36, *P* < 0.0001, respectively). Among the 8737 severe cases confirmed by laboratory testing EV71, CA16 and other enteroviruses accounted for 82.3%, 2.2% and 15.5%, respectively. Of 66 fatal cases confirmed by laboratory testing, EV71 and other enteroviruses accounted for 90.9% and 9.1%, respectively; CA16 was not detected. Although there were statistical differences observed among which viruses were associated with severe cases (χ^2^ = 216.06, *P* < 0.0001), there were no statistical differences between EV71 and other enteroviruses associated with fatal cases (χ^2^ = 6.99, *P* = 0.1362) among different years (Table 2).

### Phylogenetic analysis of the VP1 genes of EV71 and CA16

To characterize the EV71 and CA16 strains circulating in Henan and investigate their genetic origin, we analyzed the VP1 genes of viruses isolated from clinically diagnosed HFMD patient specimens. 65 EV71 and 75 CA16 viruses were isolated in our laboratory and the complete VP1 sequences of those viruses were amplified and sequenced. The nucleotide sequences of VP1 from EV71 isolates in this study were closely related to each other, sharing 95.8% to 99.9% nucleotide identity, corresponding to 98.2% to 100% amino acid identity. A similar analysis of VP1 nucleotide sequences from CA16 isolates in this study showed a 95.1% to 99.7% nucleotide identity, corresponding with a 98.1% to 100% amino acid identity.

**Figure 4:**
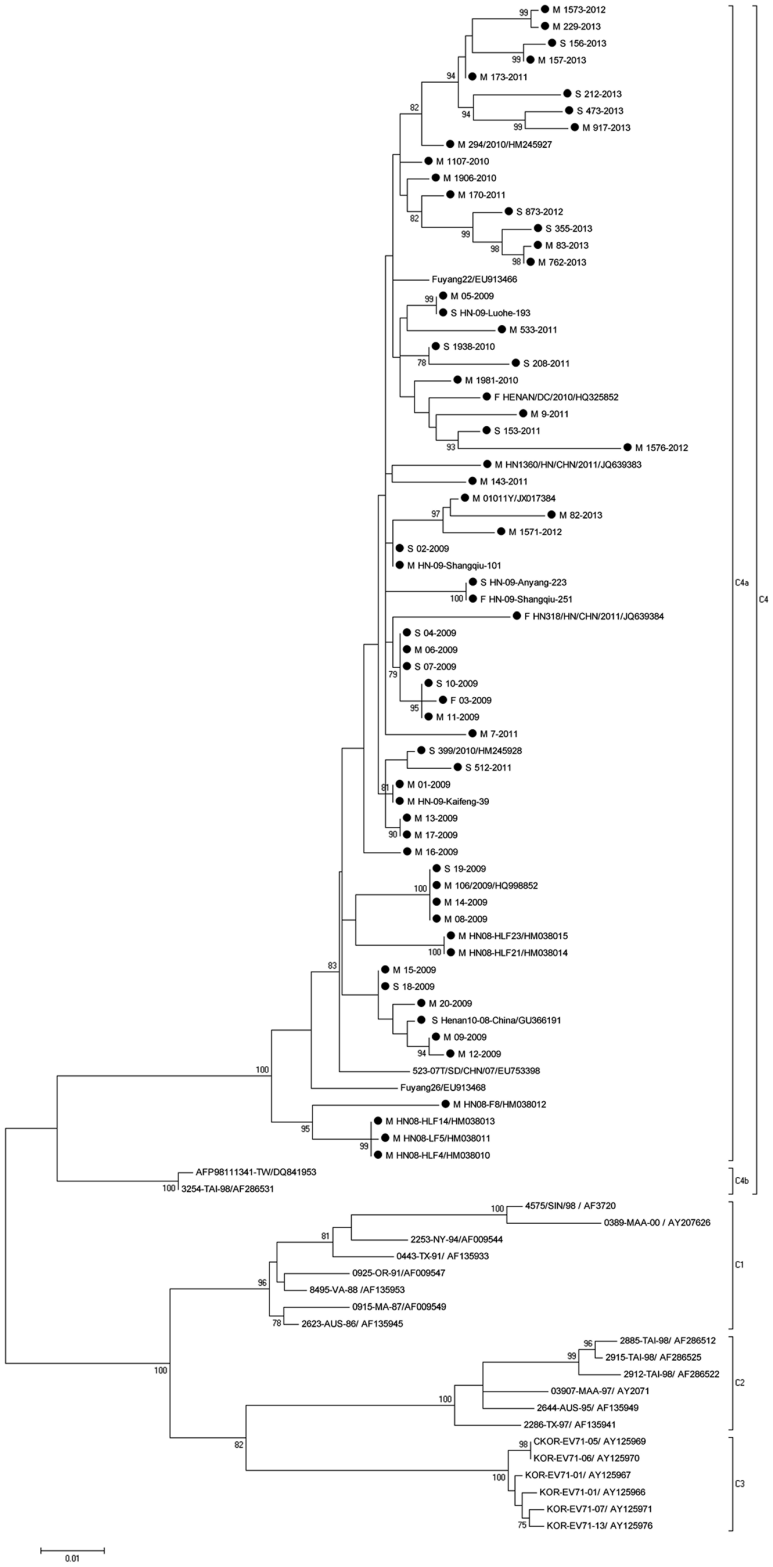
Molecular characterization and phylogenetic analyses of EV71, Henan Province. Viruses from the current study are indicated with a black dot; case severity is indicated with a M/S/F for mild, severe or fatal disease association.

**Figure 5:**
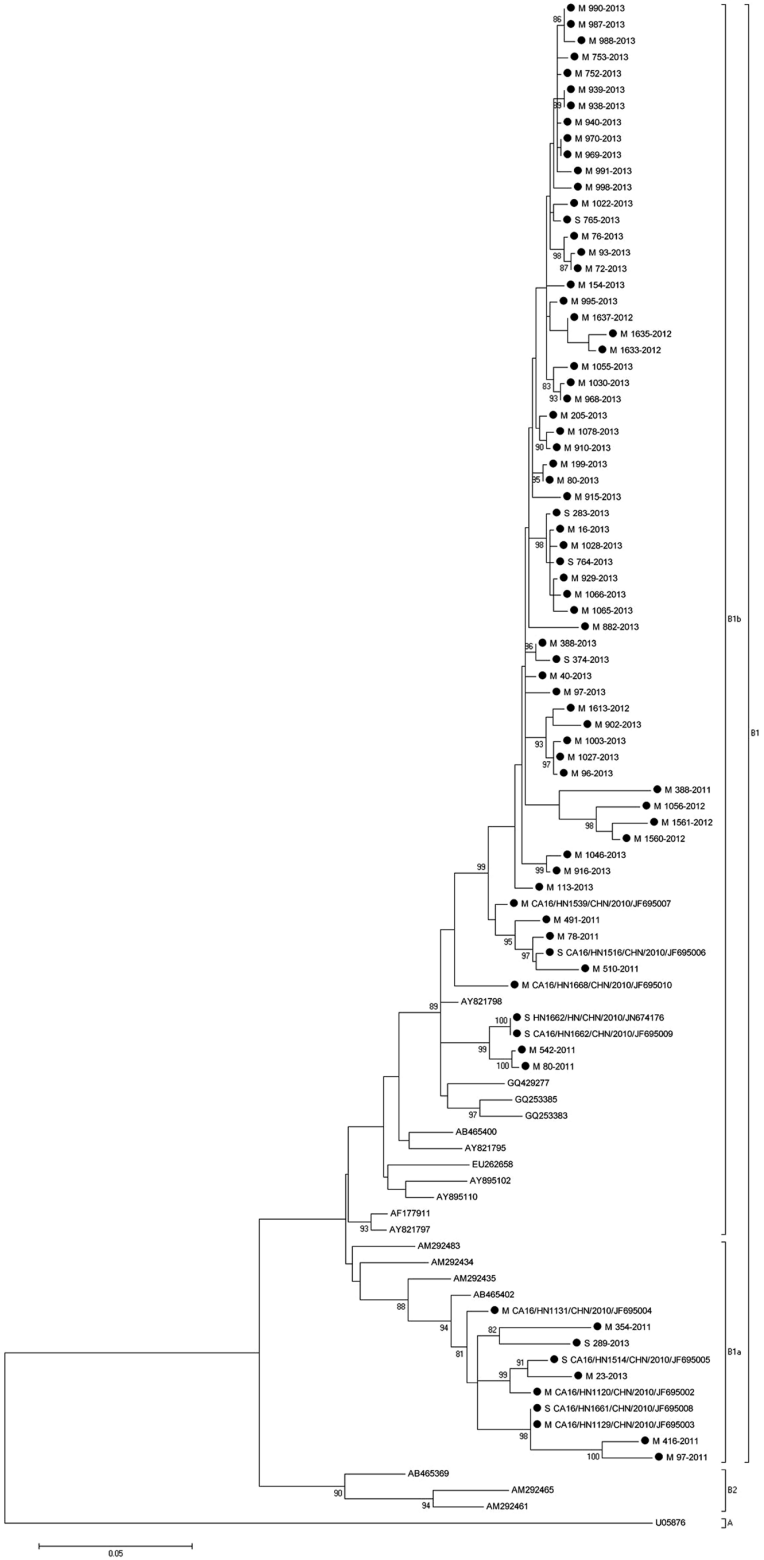
Molecular characterization and phylogenetic analyses of CA16, Henan Province. VP1 viral sequences obtained from this study are indicated with a black dot. M/S/F indicate viral association with mild, severe or fatal disease cases.

To determine the genetic characteristics of EV71 and CA16 strains circulating in their geographic location, phylogenetic analysis of these strains was based on the alignment of partial VP1 gene sequences. A total of 90 EV71 strains were used for phylogenetic analysis of the VP1 gene including the 65 EV71 strains identified in this study, All 65 EV71 strains from Henan Province clustered exclusively to genotype C, subtype 4a (C4a) (Figure 4), which was similar to EV71 sequences isolated from other provinces in mainland China. Similarly, a total of 94 CA16 strains were used for phylogenetic analysis for the VP1 gene, including the 75 CA16 strains identified in this study and 19 other CA16 strains available from the GenBank (Figure 5). The 75 CA16 viruses from Henan grouped into genotype B, subtypes 1a (B1a) and 1b (B1b). Both EV71 and CA16 viruses from this study were observed to cluster with viral sequences obtained from viruses from other provinces in China circulating at a similar time, co-evolved and co-circulated with those from surrounding provinces.

## Discussion

From January 2008 to December 2013, 400,264 HFMD cases were reported in Henan Province. The results showed that children younger than 5 years old accounted for the majority of sporadic and outbreak-associated outbreaks, indicating that this particular age group should be targeted for HFMD control and prevention. Health education efforts, including behavior change communication to prevent HFMD transmission, are recommended not only for caregivers of children at kindergartens and schools, but more widely in the community, targeting households and families with young children^2^,^7^,^14^,^20^,^21^,^22^,^23^.

Although HFMD cases in Henan Province were reported throughout the year, most were observed from April to June, suggesting that outbreaks of HFMD are seasonally-dependent. HFMD cases declined in summer and winter, suggesting that virus transmission is impacted by extremes in temperature^24^,^25^. This result also suggests that epidemiological surveillance and prevention efforts should mainly be focused on the period preceding the spring peak each year.

In the early years of the HFMD epidemic, most HFMD cases were reported in the central plains of Henan Province. Thereafter, outbreaks of HFMD spread radially, quickly spreading across the entire province. This also reflects a transmission pattern from a high population density area to other areas. In this study, Scan statistics were used to detect spatial-temporal clusters. This method has been widely used to detect space-time clusters for various diseases, including other infectious diseases and cancers^26^,^27^,^28^,^29^,^30^. Space-time cluster detection is an important tool in HFMD surveillance to identify various size, areas and duration of elevated risk^31^,^32^,^33^. Furthermore, using spatial-temporal analysis, five spatial-temporal clusters were detected, including one most likely cluster and four secondary clusters. We found the most likely cluster located in the capital city (Zhengzhou), the second-largest city (Luoyang) and a tourist-associated city (Xuchang). These cities are located on major transit centers such as highways and railways in Henan Province. This finding is consistent with previous reports that clusters are more commonly observed in areas of high population density and mobility, which also indicates that the disease may be more easily transmitted along highways and railways.

HFMD is generally a common, benign self-limiting childhood illness characterized by fever and vesicular eruption on the hands and feet and in the mouth^2^,^34^,^35^. EV71 and CA16 have been the major etiologic agents of HFMD in China since 2008. The clinical features of HFMD caused by these two viruses are indistinguishable, but EV71 infection is more commonly associated with severe neurological disease and fatalities. In contrast, CA16-associated HFMD has a milder outcome, with much lower incidence of neurological disease^36^,^37^,^38^. Previous reports are consistent with our findings. Among the 6.9% of Henan cases for which laboratory diagnosis was obtained, EV71 was the dominant pathogen: (59.53%) followed by CA16 (14.13%); more than 80% of severe cases and 90% of fatal cases were associated with EV71. Previous studies showed that all known HEV71 strains could be divided into three distinct genogroups (A, B, C) and 10 subgenogroups (B1–5, C1–5) based on VP1 gene sequences, the subgenotype C4 could be further divided into C4a and C4b clusters^39^; CA16 strains could be divided into three different clusters, called A, B, and C, however, clusters B and C in their studies correspond to sub-genotypes B2 and B1, respectively^40^. Phylogenetic analyses revealed that all 48 EV71 viruses isolated belong to the C4 sub-genotype, and all 86 CA16 strains isolated belonged to the B1 sub-genotype in this study.

HFMD epidemics have been shown to occur in 2- to 3-year cycles in the United Kingdom, Malaysia and Japan^41^,^42^,^43^. In our study, surveillance of HFMD cases showed that the highest number of cases was reported in 2009 and 2010 (approximately 100,000 cases and more than 100 per 100,000 annually), and there were approximately 60,000 cases reported in 2011, 2012 and 2013 (63.33 to 73.39 per 100,000 during 2011 to 2013), respectively. If similar cyclic epidemic patterns as observed in previous studies are to be anticipated, a new epidemic peak period will occur in Henan Province in 2014 or 2015. However, during 2009 to 2013, more than 50,000 HFMD cases were reported in Henan Province each year. This phenomenon may due to the following reasons: First, Henan is one of the most populous provinces in China with a population of about 100 million and approximately 1.2% fertility rate; 1.2 million babies are born in Henan every year. Such large number of newborns each year results in an increase in the number of susceptible individuals in the population. Second, the genotypes of EV71 or CA16 have not notably changed in the epidemic region, indicating that its virulence still remains. Third, although prevention and control measures such as environmental disinfection, health education, have been put in place to control the epidemic, they seemed not very effective to ease the epidemic situation of HFMD, probably because it was not specific targeted to the virus itself. Based on international and domestic studies, vaccination is recognized as the more effective measure for the prevention and control of this disease, especially in children under five years^44^,^45^. Our findings specially suggest that prioritization of EV71 vaccination probably is an effective public health intervention for HFMD.

This study has several limitations. First, HFMD is a self-limiting illness and may also manifest with atypical clinical symptoms, so some patients with milder or atypical symptoms were not likely included in our study. Second, the case-reporting criteria of the national guidelines for control and prevention for HFMD are based on symptoms, and only small proportion of reported HFMD cases were confirmed by laboratory testing. Thus some portion of clinically diagnosed and reported HFMD cases in our study may have in fact had other illnesses. The sensitivity and specificity of clinical diagnosis for HFMD needs to be further evaluated. Third, laboratory testing may have preferentially been ordered for more severe cases, thus potentially biasing our findings of etiologic agents. EV71 has previously been reported as associated with more severe illness, and EV71 was the most commonly identified etiologic agent among laboratory-confirmed cases in our study, but our results may have been biased by selective laboratory testing of patients with a more severe clinical presentation, or some other systematic factor distinguishing them from the bulk of clinically diagnosed cases.

## Methods

### Ethics statement

This research was approved by the Institutional Review Board at the Center for Disease Control and Prevention of Henan Province, and the methods were carried out in accordance with the principles of the Declaration of Helsinki. Written informed consents for the use of their clinical samples were obtained from all subjects (the legal guardians of the patients and contacts).

### Case definitions

HFMD case-reporting criteria are defined in the national guidelines for control and prevention for HFMD (issued by Ministry of Health in China (2009)).

#### Clinical case

For clinical diagnosis, HFMD cases include mild and severe cases. Mild case is defined as fever or not showing fever accompanied by rash (maculopapule or vesicular rash) appearing at the sites of hand, foot, mouth or buttock. Severe case is defined as having HFMD symptoms accompanied by neurological impairment, respiratory disorder or circulatory disturbance; additionally, the laboratory assay may show an increase in leukocytes in the peripheral blood, abnormal cerebrospinal fluid, increased blood glucose (GLU), and disorders as identified by electrocardiogram (ECG), myelencephalon magnetic resonance imaging, chest X-ray (CX), and ultrasound cardiogram.

#### Laboratory-confirmed case

Laboratory diagnosis was established on one of the following tests: (1) isolation of virus causing HFMD; (2) amplification of nucleic acid of EV71, CA16 or other enteroviruses; (3) neutralizing antibody titer to viruses causing HFMD (>1:256 from acute serum samples), or a four-fold elevation in the titers of neutralizing antibody in convalescent when compared to acute phase serum samples.

#### HFMD cluster

A HFMD case cluster is defined when one of the following criteria is met within one week: (1) five or more cases occurring in the same kindergarten or school; (2) two or more cases occurring in the same class or dormitory or family; (3) three or more cases occurring in the same village or community within a week.

### Clinical information and specimen collection

All HFMD cases in Henan Province were reported to the Henan Center for Disease Control and Prevention (Henan CDC) by medical practitioners from January 1, 2008 to December 31, 2013; clinical diagnosis was based on the national guidelines. At least one guardian for each patient was interviewed by CDC staff using a structured questionnaire including sociodemographic and clinical information. Data were verified by checking against medical records. Depending on the symptoms and clinical status of HFMD reported cases, appropriate clinical specimens, including a throat swab, rectal swab, or stool sample, were collected by medical workers and transported to a pathogen laboratory using cold chain (under 0°C).

### Epidemiological analysis

Case data were aggregated at the county township level. Statistical analyses were performed to describe epidemiological features, including demographic characteristics, gender and age distribution, seasonal variation, clustering and geographic distribution. Total incidence was defined as the total number of HFMD cases divided by the average population size during the study period. According to demographics and age-specific immune response to enteroviruses, we partitioned the population into five age groups: age < 1 year (infants), 1- < 3 years, 3- < 6 years, 6- < 10 years, >10 years, and age dependent analysis was performed by chi-square analysis. Time series analysis was performed to describe the seasonal distribution of HFMD cases and to detect peaks in the number of HFMD cases. The distribution of HFMD by area was analyzed separately for each municipality in Henan Province using public health geographic information system (PHGIS)(version 1.02, Chinese Center for Disease Control and Prevention, Beijing, China). All statistical analyses were performed using SAS v9.13 (SAS Institute Inc., Cary, NC). An association at the *P* < 0.05 level was considered statistically significant.

### Spatio-temporal cluster analysis

A space-time scan statistic was used to determine the presence of space-time clusters of HFMD cases during the study period. A retrospective space-time scan statistic was applied to detect high risk clusters of HFMD cases using the SaTScanTM software (version 9.1, Boston, MA, USA) with a discrete Poisson model. The 168 counties within Henan Province were used as spatial units, covering the 6 years from January 2008 to December 2013 as the time unit. In order to scan clusters of any size, the largest radius was set to 50% of the total population at risk with a circular scanning window, and the largest height was set to 50% of the total study period. To ensure sufficient statistical power and taking into account the time required to conduct these computations, 999 Monte Carlo replications were set, and clusters with statistical significance of *P* < 0.05 were all reported, including secondary clusters that did not overlap with a previously reported cluster. Among the statistically significant clusters, a cluster with a maximum log likelihood ratio (LLR) was least likely to have occurred by chance, and is therefore a bona fide cluster. Secondary clusters were those in rank order after the most likely cluster, based on their likelihood ratio test statistic. Furthermore, we used PHGIS to visualize the results of scan statistic analysis.

### Detection of viral RNA from clinical specimens

Total RNA was directly extracted from clinical specimens using a QIAamp viral RNA mini Kit (Qiagen, Hilden, Germany) according to the manufacturer's instructions. RNA was eluted in a final volume of 60 μL elution buffer and used immediately or stored at −80°C. RNA from each sample was examined by conventional RT-PCR or real-time RT-PCR.

Clinical specimens collected from 2008 to 2009 were examined by conventional RT-PCR. Conventional RT-PCR was conducted using a Quant One Step RT-PCR Kit (Tiangen Biotech, Beijing, China) containing specific primers for EV71, CA16, and pan-enterovirus, respectively. The clinical specimens collected from 2010 to 2013 were examined using commercially available real-time RT-PCR kits (Diagnostic Kit for Human Enterovirus, EV71 and CA16, Beijing Kinghawk Pharmaceutical Co., Ltd, Beijing, China or Jiangshu Shuoshi Biological Technology Co., Ltd, Taizhou, China) as per the manufacturer's protocols. Test results were classified into four categories: enterovirus negative, EV71 positive, CA16 positive, or positive for another enterovirus without further serotype identification.

### Virus isolation and sequencing

After processing, specimens from clinically diagnosed HFMD patients were inoculated into a 25-cm^2^ flask containing confluent rhabdomyosarcoma (RD) cells monolayers. Tubes were incubated at 36°C and observed on the 3rd, 5th and 7th days post-inoculation. Virus was harvested from tubes when the cytopathic effect (CPE) affected 75 ~ 100% of the monolayer. If no CPE was observed in the initial RD cell culture, supernatants were collected and inoculated into a new RD cell culture flask for up to three passages. Recovered viral particles were identified using RT-PCR methods and the complete VP1 gene sequences from EV71 and CA16 isolates was amplified as previously described^46^. Amplification products were purified using a QIAquick Gel Extraction Kit (Qiagen, Hilden, Germany), and sent to Sangon Biotech Co., Ltd (Shanghai, China) for DNA sequencing using an automated ABI 3730 DNA sequencer.

### Phylogenetic analyses

Molecular phylogenetic analysis was conducted using the maximum likelihood (ML) method based on the Kimura 2-parameter model in the MEGA 5 software^47^. The tree with the highest log likelihood was shown. The percentage of trees in which the associated taxa clustered together was shown next to the branches. Initial tree(s) for the heuristic search were obtained automatically as follows: When the number of common sites was <100 or <1/4 of the total number of sites, the Maximum Parsimony method was used; otherwise the BIONJ method with MCL distance matrix was used. Trees were drawn to scale, with branch lengths measured in the number of substitutions per site. All available nucleotide sequences of complete VP1 genes from EV71 or CA16 identified in GenBank were analyzed, together with the VP1 genes obtained in this study (Supplementary Table 2), and phylogenetic trees were constructed in order to understand the evolutionary relationships among EV71 and CA16 isolates.

## Author Contributions

X.H., S.W., J.K. and B.X. conceived the study and drafted the paper, H.W., Y.D., Y.X. and X.L. performed the experiments, L.L., J.S., H.W., Y.W., G.L. and W.C. gathered and analyzed the data. All authors reviewed the manuscript.

## Supplementary Material

Supplementary Information Supplementary information

## Figures and Tables

**Table 1:** Numbers of reported hand, foot and mouth disease cases by gender, residence, age group and year

Variable	2008	2009	2010	2011	2012	2013
**HFMD Reported Cases**						
Mild cases	11816	97502	87813	55839	64962	59882
Severe cases	10	3932	8937	3692	3923	1815
Deaths	0	46	48	23	11	13
Total(Incidence rate/10^5^)	11826 (12.63)	101480 (107.63)	96798 (102.03)	59554 (63.33)	68896 (73.39)	61710 (65.61)
**Gender**						
Male (Incidence rate/10^5^)	7877 (16.35)	64953 (133.87)	62284 (127.62)	38682 (81.44)	44300 (91.10)	38134 (78.27)
Female (Incidence rate/10^5^)	3949 (8.69)	36527 (79.81)	34514 (74.92)	20872 (44.85)	24596 (54.36)	23576 (52.00)
Gender ratio	1.99	1.78	1.80	1.85	1.80	1.62
**Residence**						
Scattered children	9015	83460	79053	48099	56321	51579
Kindergarten children	2366	15633	15643	10396	11326	8987
School students	395	2191	1938	951	1136	1019
Other	50	196	164	108	113	125
**Age Group (%)**						
<1	802 (6.78%)	8284 (8.16%)	7596 (7.85%)	4988 (8.38%)	5781 (8.39%)	6404 (10.38%)
1- < 3	6686 (56.54%)	59749 (58.88%)	63533 (65.63%)	39090 (65.64%)	45189 (65.59%)	40023 (64.86%)
3- < 5	3261 (27.57%)	26557 (26.17%)	20518 (21.20%)	12758 (21.42%)	14675 (21.30%)	12377 (20.06%)
5- < 10	939 (7.94%)	6033 (5.95%)	4417 (4.56%)	2350 (3.94%)	2842 (4.13%)	2478 (4.02%)
≥10	138 (1.17%)	857 (0.84%)	734 (0.76%)	368 (0.62%)	409 (0.59%)	428 (0.68%)
**Reports of Case Clusters**						
Villages (No. of cases)	81 (291)	964 (3715)	941 (3010)	66 (219)	136 (448)	132(427)
Communities (No. of cases)	16 (65)	387 (1427)	147 (485)	8 (28)	31 (99)	42(142)
kindergarten and schools (No. of cases)	4 (24)	269 (1748)	165 (1197)	106 (694)	174 (868)	195(1054)
Families (No. of cases)	4 (9)	187 (406)	207 (451)	68 (143)	107 (226)	138(308)
Total	105 (379)	1807 (7296)	1460 (5143)	248 (1084)	448 (1641)	507(1931)

Scattered children: a group of children who are not currently in school or kindergarten, but dwelling in scattered areas.

Kindergarten children: a group of children between the ages of three and six, who receive preschool education in a kindergarten.

School students: a group of people who studying in the school, including primary school, secondary school and college.

**Table 2:** Etiological composition of laboratory-confirmed cases, Henan Province, 2008 to 2013

Year	EV71			CA16			Other enteroviruses			Total
	Mild cases	Severe cases	Deaths	Mild cases	Severe cases	Deaths	Mild cases	Severe cases	Deaths	
2008	185	5	0	27	0	0	46	0	0	263
2009	2125	1476	31	814	11	0	1631	290	1	6379
2010	1823	3258	10	354	48	0	1127	667	3	7290
2011	1485	932	8	836	63	0	687	129	2	4142
2012	2170	1128	5	825	42	0	1083	130	0	5383
2013	1448	389	6	865	29	0	1358	140	0	4235
Total	9236	7188	60	3721	193	0	5932	1356	6	27692
