# Supplementary material for: Epidemiological and Etiological Characteristics of Hand, Foot, and Mouth Disease in Henan, China, 2008–2013
Source: Sci Rep. 2015 Mar 10;5:8904. doi: 10.1038/srep08904 (PMC4354091; doi:10.1038/srep08904)
Supplement: Supplementary Information [file srep08904-s1.pdf]

# Epidemiological and Etiological Characteristics of Hand, Foot, and Mouth Disease in Henan, China, 2008-2013

Xueyong Huang[1,2]*, Haiyan Wei[1]*, Shuyu Wu[3,4], Yanhua Du[1,2], Licheng Liu[5,6], Jia Su[1], Yuling Xu[1], Haifeng Wang[1], Xingle Li[1,2], Yanxia Wang[1], Guohua Liu[1], Weijun Chen[5,6], John David Klena [3,4], Bianli Xu[1]

[1]Henan Center for Disease Control and Prevention, Zhengzhou, People's Republic of China

[2]Henan Key Laboratory of pathogenic microorganisms, Zhengzhou, People's Republic of China

[3]International Emerging Infections Program, US Centers for Disease Control and Prevention, Beijing, People's Republic of China

[4]Global Disease Detection Branch, Division of Global Health Protection, Center for Global Health, Centers for Disease Control and Prevention, Atlanta, USA

[5]State Key Laboratory of Pathogens and Biosecurity, Institute of Microbiology and Epidemiology, Academy of Military Medical Sciences, Beijing, People's Republic of China

[6]Key Laboratory of Genome Sciences and Information, Beijing Institute of Genomics, Chinese Academy of Sciences, Beijing, People's Republic of China.

Supplementary Table 1. The GenBank accession numbers of SFTSV obtained in this study

| Name of strain | GenBank accession No. | Isolation source | Geographic origin | Clinical manifestation | Year of isolation | Enterovirus serotype |
|---|---|---|---|---|---|---|
| Henan10-08-China | GU366191 | stool | Zhengzhou | Severe case | 2008 | EV71 |
| HN08-F8 | HM038012 | stool | Xinxiang | Mild case | 2008 | EV71 |
| HN08-HLF21 | HM038014 | stool | Xinxiang | Mild case | 2008 | EV71 |
| HN08-HLF23 | HM038015 | stool | Xinxiang | Mild case | 2008 | EV71 |
| HN08-HLF4 | HM038010 | stool | Xinxiang | Mild case | 2008 | EV71 |
| HN08-LF5 | HM038011 | stool | Xinxiang | Mild case | 2008 | EV71 |
| 03-2009 | KM288732 | stool | Zhumadian | Fatal case | 2009 | EV71 |
| 06-2009 | KM288735 | stool | Zhumadian | Mild case | 2009 | EV71 |
| 07-2009 | KM288736 | stool | Zhumadian | Severe case | 2009 | EV71 |
| 08-2009 | KM288737 | stool | Zhumadian | Mild case | 2009 | EV71 |
| 01-2009 | KM288730 | stool | Zhoukou | Mild case | 2009 | EV71 |
| 02-2009 | KM288731 | stool | Zhoukou | Severe case | 2009 | EV71 |
| 04-2009 | KM288733 | stool | Zhengzhou | Severe case | 2009 | EV71 |
| 05-2009 | KM288734 | stool | Zhengzhou | Mild case | 2009 | EV71 |
| 17-2009 | KM288746 | stool | Xinyang | Mild case | 2009 | EV71 |
| 18-2009 | KM288747 | stool | Xinyang | Severe case | 2009 | EV71 |
| 19-2009 | KM288748 | stool | Xinyang | Severe case | 2009 | EV71 |
| 20-2009 | KM288749 | stool | Xinyang | Mild case | 2009 | EV71 |
| HN-09-Shangqiu-101 | KM288753 | stool | Shangqiu | Mild case | 2009 | EV71 |
| HN-09-Shangqiu-251 | KM288754 | stool | Shangqiu | Fatal case | 2009 | EV71 |
| 15-2009 | KM288744 | stool | Puyang | Severe case | 2009 | EV71 |
| 16-2009 | KM288745 | stool | Puyang | Mild case | 2009 | EV71 |
| HN-09-Luohe-193 | KM288752 | stool | Luohe | Severe case | 2009 | EV71 |
| 09-2009 | KM288738 | stool | Kaifeng | Mild case | 2009 | EV71 |
| 10-2009 | KM288739 | stool | Kaifeng | Severe case | 2009 | EV71 |
| 11-2009 | KM288740 | stool | Kaifeng | Mild case | 2009 | EV71 |
| EV71-106/2009 | HQ998852 | stool | Kaifeng | Mild case | 2009 | EV71 |
| HN-09-Kaifeng-39 | KM288751 | stool | Kaifeng | Mild case | 2009 | EV71 |
| 12-2009 | KM288741 | stool | Jiaozuo | Mild case | 2009 | EV71 |
| 13-2009 | KM288742 | stool | Jiaozuo | Mild case | 2009 | EV71 |
| 14-2009 | KM288743 | stool | Jiaozuo | Mild case | 2009 | EV71 |
| HN-09-Anyang-223 | KM288750 | stool | Anyang | Severe case | 2009 | EV71 |
| HENAN/DC/2010 | HQ325852 | stool | Zhengzhou | Fatal case | 2010 | EV71 |
| 1938-2010 | KM260010 | stool | Xinxiang | Severe case | 2010 | EV71 |
| 1906-2010 | KM260009 | stool | Sanmenxia | Mild case | 2010 | EV71 |
| EV71-294/2010 | HM245927 | stool | Pingdingshan | Mild case | 2010 | EV71 |
| 1107-2010 | KM260008 | stool | Luohe | Mild case | 2010 | EV71 |
| 1981-2010 | KM260011 | stool | Kaifeng | Mild case | 2010 | EV71 |
| EV71-399/2010 | HM245928 | stool | Kaifeng | Severe case | 2010 | EV71 |
| CA16/HN1668/CHN/2010 | JF695010 | stool | Kaifeng | Mild case | 2010 | CA16 |

| | | | | | | |
|---|---|---|---|---|---|---|
| CA16/HN1129/CHN/2010 | JF695003 | stool | Luoyang | Mild case | 2010 | CA16 |
| CA16/HN1131/CHN/2010 | JF695004 | stool | Luoyang | Mild case | 2010 | CA16 |
| CA16/HN1661/CHN/2010 | JF695008 | stool | Nanyang | Severe case | 2010 | CA16 |
| CA16/HN1662/CHN/2010 | JF695009 | stool | Nanyang | Severe case | 2010 | CA16 |
| HN1662/HN/CHN/2010 | JN674176 | stool | Nanyang | Severe case | 2010 | CA16 |
| CA16/HN1514/CHN/2010 | JF695005 | stool | Zhengzhou | Severe case | 2010 | CA16 |
| CA16/HN1516/CHN/2010 | JF695006 | stool | Zhengzhou | Severe case | 2010 | CA16 |
| CA16/HN1539/CHN/2010 | JF695007 | stool | zhoukou | Mild case | 2010 | CA16 |
| CA16/HN1120/CHN/2010 | JF695002 | stool | Zhumadian | Mild case | 2010 | CA16 |
| HN1360/HN/CHN/2011 | JQ639383 | stool | Hebi | Mild case | 2011 | EV71 |
| 7-2011 | KM260012 | stool | kaifeng | Mild case | 2011 | EV71 |
| 9-2011 | KM260013 | stool | kaifeng | Mild case | 2011 | EV71 |
| 143-2011 | KM260014 | stool | Luoyang | Mild case | 2011 | EV71 |
| 208-2011 | KM260018 | stool | Luohe | Severe case | 2011 | EV71 |
| 512-2011 | KM260019 | stool | Luohe | Severe case | 2011 | EV71 |
| 173-2011 | KM260017 | stool | Sanmenxia | Mild case | 2011 | EV71 |
| 533-2011 | KM260020 | stool | Sanmenxia | Mild case | 2011 | EV71 |
| 170-2011 | KM260016 | stool | Shangqiu | Mild case | 2011 | EV71 |
| 153-2011 | KM260015 | stool | Zhengzhou | Severe case | 2011 | EV71 |
| HN318/HN/CHN/2011 | JQ639384 | stool | Zhengzhou | Fatal case | 2011 | EV71 |
| EV71-01011Y | JX017384 | stool | Zhengzhou | Mild case | 2011 | EV71 |
| 542-2011 | KM260056 | stool | Zhengzhou | Mild case | 2011 | CA16 |
| 354-2011 | KM260061 | stool | Xinxiang | Mild case | 2011 | CA16 |
| 78-2011 | KM260064 | stool | Xinxiang | Mild case | 2011 | CA16 |
| 80-2011 | KM260063 | stool | Xinxiang | Mild case | 2011 | CA16 |
| 97-2011 | KM260062 | stool | Xinxiang | Mild case | 2011 | CA16 |
| 388-2011 | KM260060 | stool | Nanyang | Mild case | 2011 | CA16 |
| 510-2011 | KM260057 | stool | Luohe | Mild case | 2011 | CA16 |
| 416-2011 | KM260059 | stool | Luoyang | Mild case | 2011 | CA16 |
| 491-2011 | KM260058 | stool | Kaifeng | Mild case | 2011 | CA16 |
| 873-2012 | KM260021 | stool | Pingdingshan | Severe case | 2012 | EV71 |
| 1571-2012 | KM260022 | stool | Shangqiu | Mild case | 2012 | EV71 |
| 1573-2012 | KM260023 | stool | Shangqiu | Mild case | 2012 | EV71 |
| 1576-2012 | KM260024 | stool | Shangqiu | Mild case | 2012 | EV71 |
| 1056-2012 | KM260071 | stool | Nanyang | Mild case | 2012 | CA16 |
| 1633-2012 | KM260067 | stool | Luohe | Mild case | 2012 | CA16 |
| 1635-2012 | KM260066 | stool | Luohe | Mild case | 2012 | CA16 |
| 1637-2012 | KM260065 | stool | Luohe | Mild case | 2012 | CA16 |
| 1613-2012 | KM260068 | stool | Hebi | Mild case | 2012 | CA16 |
| 1560-2012 | KM260070 | stool | Anyang | Mild case | 2012 | CA16 |
| 1561-2012 | KM260069 | stool | Anyang | Mild case | 2012 | CA16 |
| 762-2013 | KM260033 | stool | Zhengzhou | Mild case | 2013 | EV71 |
| 355-2013 | KM260031 | stool | Xinxiang | Severe case | 2013 | EV71 |
| 229-2013 | KM260030 | stool | Shangqiu | Mild case | 2013 | EV71 |

| | | | | | | |
|---|---|---|---|---|---|---|
| 917-2013 | KM260034 | stool | Shangqiu | Mild case | 2013 | EV71 |
| 473-2013 | KM260032 | stool | Luohe | Severe case | 2013 | EV71 |
| 156-2013 | KM260027 | stool | Luoyang | Severe case | 2013 | EV71 |
| 157-2013 | KM260028 | stool | Luoyang | Mild case | 2013 | EV71 |
| 82-2013 | KM260025 | stool | Kaifeng | Mild case | 2013 | EV71 |
| 83-2013 | KM260026 | stool | Kaifeng | Mild case | 2013 | EV71 |
| 212-2013 | KM260029 | stool | Hebi | Severe case | 2013 | EV71 |
| 902-2013 | KM260098 | stool | zhoukou | Mild case | 2013 | CA16 |
| 910-2013 | KM260097 | stool | zhoukou | Mild case | 2013 | CA16 |
| 1022-2013 | KM260080 | stool | Zhengzhou | Mild case | 2013 | CA16 |
| 764-2013 | KM260101 | stool | Zhengzhou | Severe case | 2013 | CA16 |
| 765-2013 | KM260100 | stool | Zhengzhou | Severe case | 2013 | CA16 |
| 1055-2013 | KM260075 | stool | Xuchang | Mild case | 2013 | CA16 |
| 1065-2013 | KM260074 | stool | Xuchang | Mild case | 2013 | CA16 |
| 1066-2013 | KM260073 | stool | Xuchang | Mild case | 2013 | CA16 |
| 16-2013 | KM260120 | stool | Xuchang | Mild case | 2013 | CA16 |
| 283-2013 | KM260107 | stool | Xuchang | Severe case | 2013 | CA16 |
| 1003-2013 | KM260081 | stool | Xinyang | Mild case | 2013 | CA16 |
| 1078-2013 | KM260072 | stool | Xinxiang | Mild case | 2013 | CA16 |
| 1046-2013 | KM260076 | stool | Shangqiu | Mild case | 2013 | CA16 |
| 113-2013 | KM260111 | stool | Shangqiu | Mild case | 2013 | CA16 |
| 915-2013 | KM260096 | stool | Shangqiu | Mild case | 2013 | CA16 |
| 916-2013 | KM260095 | stool | Shangqiu | Mild case | 2013 | CA16 |
| 289-2013 | KM260106 | stool | Puyang | Severe case | 2013 | CA16 |
| 23-2013 | KM260119 | stool | Pingdingshan | Mild case | 2013 | CA16 |
| 882-2013 | KM260099 | stool | Pingdingshan | Mild case | 2013 | CA16 |
| 96-2013 | KM260113 | stool | Nanyang | Mild case | 2013 | CA16 |
| 97-2013 | KM260112 | stool | Nanyang | Mild case | 2013 | CA16 |
| 154-2013 | KM260110 | stool | Luoyang | Mild case | 2013 | CA16 |
| 40-2013 | KM260118 | stool | Luoyang | Mild case | 2013 | CA16 |
| 199-2013 | KM260109 | stool | Kaifeng | Mild case | 2013 | CA16 |
| 72-2013 | KM260117 | stool | Kaifeng | Mild case | 2013 | CA16 |
| 76-2013 | KM260116 | stool | Kaifeng | Mild case | 2013 | CA16 |
| 80-2013 | KM260115 | stool | Kaifeng | Mild case | 2013 | CA16 |
| 929-2013 | KM260094 | stool | Kaifeng | Mild case | 2013 | CA16 |
| 93-2013 | KM260114 | stool | Kaifeng | Mild case | 2013 | CA16 |
| 995-2013 | KM260083 | stool | Kaifeng | Mild case | 2013 | CA16 |
| 998-2013 | KM260082 | stool | Kaifeng | Mild case | 2013 | CA16 |
| 374-2013 | KM260105 | stool | Jiyuan | Severe case | 2013 | CA16 |
| 388-2013 | KM260104 | stool | Jiyuan | Mild case | 2013 | CA16 |
| 1027-2013 | KM260079 | stool | Hebi | Mild case | 2013 | CA16 |
| 1028-2013 | KM260078 | stool | Hebi | Mild case | 2013 | CA16 |
| 1030-2013 | KM260077 | stool | Hebi | Mild case | 2013 | CA16 |
| 205-2013 | KM260108 | stool | Hebi | Mild case | 2013 | CA16 |

| | | | | | | |
|---|---|---|---|---|---|---|
| 968-2013 | KM260090 | stool | Hebi | Mild case | 2013 | CA16 |
| 969-2013 | KM260089 | stool | Hebi | Mild case | 2013 | CA16 |
| 970-2013 | KM260088 | stool | Hebi | Mild case | 2013 | CA16 |
| 752-2013 | KM260103 | stool | Anyang | Mild case | 2013 | CA16 |
| 753-2013 | KM260102 | stool | Anyang | Mild case | 2013 | CA16 |
| 938-2013 | KM260093 | stool | Anyang | Mild case | 2013 | CA16 |
| 939-2013 | KM260092 | stool | Anyang | Mild case | 2013 | CA16 |
| 940-2013 | KM260091 | stool | Anyang | Mild case | 2013 | CA16 |
| 987-2013 | KM260087 | stool | Anyang | Mild case | 2013 | CA16 |
| 988-2013 | KM260086 | stool | Anyang | Mild case | 2013 | CA16 |
| 990-2013 | KM260085 | stool | Anyang | Mild case | 2013 | CA16 |
| 991-2013 | KM260084 | stool | Anyang | Mild case | 2013 | CA16 |

Supplementary Table 2. Spatial-temporal clusters of HFMD, Henan Province, 2008 to 2013

| Cluster period | Cluster center /Radius | Number of counties in the cluster | Observed cases | Expected cases | Relative risk | P-value |
|---|---|---|---|---|---|---|
| 2009-2010 | (34.46 N, 113.05 E) / 92.94 km | 54 | 78431 | 33384.86 | 2.68 | < 0.001 |
| 2010 | (32.98 N, 113.99E) / 98.61 km | 26 | 22162 | 12196.66 | 1.86 | < 0.001 |
| 2009 | (34.45 N, 115.07 E) / 79.67 km | 19 | 13992 | 8979.75 | 1.58 | < 0.001 |
| 2009-2010 | (35.90 N, 114.19 E) / 32.06 km | 10 | 8989 | 2917.46 | 3.13 | < 0.001 |
| 2009-2010 | (35.86 N, 115.51E) / 44.74 km | 6 | 8687 | 3527.12 | 2.50 | < 0.001 |
